# Performance of Forest Bryophytes with Different Geographical Distributions Transplanted across a Topographically Heterogeneous Landscape

**DOI:** 10.1371/journal.pone.0112943

**Published:** 2014-11-11

**Authors:** C. Johan Dahlberg, Johan Ehrlén, Kristoffer Hylander

**Affiliations:** Department of Ecology, Environment and Plant Sciences, Stockholm University, Stockholm, Sweden; Portland State University, United States of America

## Abstract

Most species distribution models assume a close link between climatic conditions and species distributions. Yet, we know little about the link between species' geographical distributions and the sensitivity of performance to local environmental factors. We studied the performance of three bryophyte species transplanted at south- and north-facing slopes in a boreal forest landscape in Sweden. At the same sites, we measured both air and ground temperature. We hypothesized that the two southerly distributed species *Eurhynchium angustirete* and *Herzogiella seligeri* perform better on south-facing slopes and in warm conditions, and that the northerly distributed species *Barbilophozia lycopodioides* perform better on north-facing slopes and in relatively cool conditions. The northern, but not the two southern species, showed the predicted relationship with slope aspect. However, the performance of one of the two southern species was still enhanced by warm temperatures. An important reason for the inconsistent results can be that microclimatic gradients across landscapes are complex and influenced by many climate-forcing factors. Therefore, comparing only north- and south-facing slopes might not capture the complexity of microclimatic gradients. Population growth rates and potential distributions are the integrated results of all vital rates. Still, the study of selected vital rates constitutes an important first step to understand the relationship between population growth rates and geographical distributions and is essential to better predict how climate change influences species distributions.

## Introduction

The niche of a species can be thought of as the environmental conditions where population growth rate is positive [1]–[3]. Population growth rate is determined by the vital rates (survival, growth and reproduction) of individuals, which in turn are influenced by the environmental conditions [4]–[6]. The potential geographic distribution of a species will thus be determined by the environmental conditions that influence vital rates, i.e. the performance of individuals. Among the environmental variables that have rendered most focus in recent work on species distributions are various climatic conditions. Most species distribution models are based on the presence or absence of species and use coarse scale climatic variables [7], [8]. However, we still know little about how population growth rates and the performance of individuals depend on local climatic conditions [8]–[10]. Yet an improved understanding of individual and population responses to local climatic conditions across species ranges should enhance our ability to predict the response of species to future climate change.

The density of many species is lower towards its range margins due to an overall lower quality in habitat or more sparse distribution of suitable habitat patches [11], [12]. This implies that populations towards their range margins might become more restricted to habitat patches with locally favorable microclimate [13], [14]. According to this reasoning, differences in performance that relate to variation in local climate should be more accentuated towards the range margins. In fact, the presence of species at favorable microclimatic sites far away from their main distributions is one of the strongest cases for climate as a regulating factor for species distributions [12]. Climate has been assumed to directly influence species distributions, especially towards cold or dry conditions. In such situations, the asymmetric abiotic stress limitation (AASL) hypothesis predicts the occurrence of a species to be limited by its physiological tolerance due to stressful abiotic factors [15]. This hypothesis is based on the assumption that the physiological tolerance of individuals influence their fitness, and thus the distribution of species [6], [16]. Consequently, studies of the relationship between individual performance and climate variables at species range margins are essential to understand how climate regulates distribution patterns, and how climate change will lead to future range shifts.

There is often a relatively weak correlation between the local climate, which organisms experience, and the regional climate [14]. Large altitudinal variation and complex terrain can lead to considerable variation in microclimate across a landscape [14], [17], [18]. One factor leading to microclimatic variation is aspect; mountain slopes facing the equator receive more incoming solar radiation than slopes facing the poles [19]–[21]. Therefore, equator-ward slopes are often both warmer and drier and have larger temperature fluctuations than pole-ward slopes [22]–[24]. As a consequence, north- and south-facing slopes are potentially good candidates for studies on microclimatic gradients and have also shown to influence species composition [25]. However, the interactions of many climate-forcing factors (e.g. distance to sea, canopy cover) can lead to complex microclimatic patterns and obscure the climate regulating role of aspect [20], [26]. Thus, to understand the role of climate for individual performance, we need to address the local scale where microclimatic conditions correspond to the limits of a species realized niche.

The aim with this study was to explore how performance of individuals in three bryophyte species with different geographical distributions is influenced by local variation in microclimate across a topographic heterogeneous landscape. Bryophytes are particularly suitable for studies of performance and distribution patterns in relation to microclimatic gradients for several reasons. First, they closely depend on the microclimate of their immediate surroundings due to their small size and poikilohydric state; the latter resulting in that tissue water content is similar to the surrounding environment [27]–[30]. Secondly, bryophytes are common and occur in a broad range of habitats worldwide, often dominating the ground cover in boreal and boreonemoral forests [31]–[33]. Finally, many bryophyte species are easy to transplant, making them an ideal experimental system [34], [35]. Despite these advantages, bryophytes remain relatively underrepresented in ecological studies.

We investigated the relationships between individual performance of three transplanted bryophyte species, in terms of growth, vitality and capsule maturation, and environmental conditions, in terms of local temperature and slope aspect. We hypothesized that the performance of transplants would reflect the general distribution pattern of each species. Two southerly distributed species (*Eurhynchium angustirete* and *Herzogiella seligeri*) were therefore hypothesized to perform better at south-facing slopes and in warm conditions, whereas a northerly distributed species (*Barbilophozia lycopodioides*) was hypothesized to perform better at north-facing slopes and in relatively cool microclimatic positions.

## Materials and Methods

### Study area

**Figure 1:**
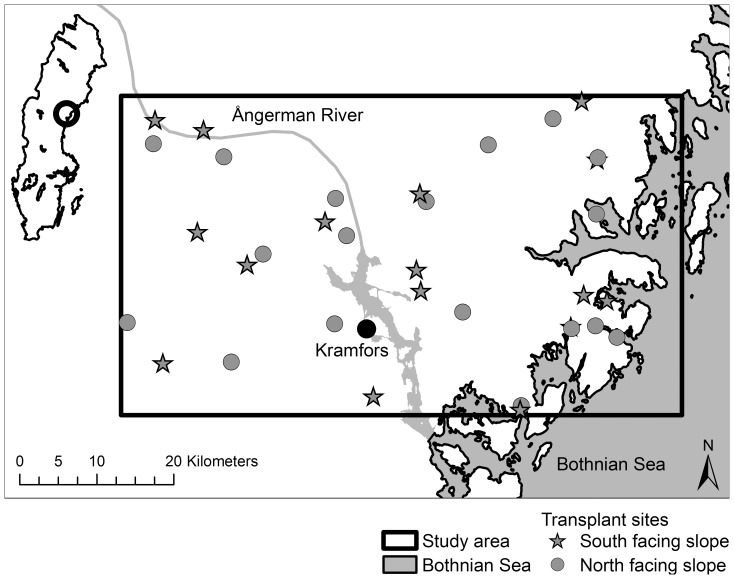
Location of the study area (rectangle) and the 35 transplant sites in the middle of Sweden. Circles indicate north-facing slopes and stars indicate south-facing slopes. Background overview maps: © Lantmäteriet Gävle 2012 (Permit i2012/899).

The study area is located in central Sweden adjacent to the Bothnian sea in the county of Ångermanland (Figure 1; between the latitudes 62°50′ and 63°12′N). It extends 73 km from east to west and 42 km from north to south.

The area is characterized by hills reaching heights of about 100 to 200 meters above the valley bottoms. The altitude reaches 150–300 m a.s.l. by the coast and increases to 350–470 m a.s.l. further inland. The soil material is sandy/loamy/silty tills forming mainly podzolic soils on top of bedrock of mostly gneiss. The mean temperature for July is 15.6°C and the annual mean precipitation 671 mm by the city of Kramfors (mean values from 1961–1990; [36]). The area belongs phytogeographically mainly to the middle boreal subzone, and by the coast to the southern boreal subzone [32], [37]. In these subzones, many species have their northern range margin and are found at sites with locally favorable conditions, e.g. on south-facing mountain slopes [38], [39].

### Study design and data collection

The sites were selected with the help of local floras [39], [40], aerial photos, topographic maps and field visits. We selected 15 south-facing and 18 north-facing slopes (in total 33 slopes), stratified according to distance to the sea. Each slope should possess forest of at least 50 years of age (often much more), dominated or co-dominated by Norway spruce (*Picea abies*), with more or less closed canopy cover and positioned on mesic soil. In such forests, we selected a 4×4 meter site on each slope. Sites were stratified for variation in altitude, and selected to be situated at a minimum of 10 meters from younger forest stands, open ground and streams that could influence the microclimate. For the same reason, near vicinity to rocky outcrops was avoided. Most of the sites were situated in forests solely dominated by *P. abies*, but at some sites birch (*Betula* spp.) or Scots pine (*Pinus sylvestris*) co-dominated the tree layer together with *P. abies*.

We transplanted three species to all selected sites. The two mosses *Herzogiella seligeri* and *Eurhynchium angustirete* possess a southern distribution in Sweden and are both common south of the river Dalälven, but *H. seligeri* also occur along the coast northward into the focal study landscape. The foliose liverwort *Barbilophozia lycopodioides* is common north of the river Dalälven and thus occupies a more northern distribution in Sweden, but it also has scattered occurrences in southern Sweden [40], [41]. We collected the transplant material in April 2011 from localities within the main ranges of the species. Material of *H. seligeri* and *E. angustirete* was gathered in the hemiboreal subzone [32], [37] 330 km south of the transplantation experiment (Uppland, Erken, Lat 59°50′6.30″; Long 18°30′15.03″). The growing season is on average 20 days longer at this collection site compared to the study area, while the precipitation is similar ([36], [42]). *Eurhynchium angustirete* grew on mesic soil in a flat spruce forest with vegetation dominated by low herbs, whereas *H. seligeri* grew on decaying logs of spruce in the same forest. Material of *B. lycopodioides* was collected in a spruce forest on a north-facing slope within the same landscape as the experiment (Ångermanland, Latberget; Lat 62°56′4.96″; Long 17°42′19.74″). When gathered, both *E. angustirete* and *B. lycopodioides* were carefully removed from the soil while for *H. seligeri* pieces of wood were retained for all transplants. Before transplanting, the material was stored in carpets on moist forest floor in the study area, sheltered from direct sunlight.

At the start of the experiment in April to May 2011, we removed the vegetation down to the topsoil and planted a total of nine transplants (three transplants per species) at each site. We placed the transplants directly on the ground and attached them using thin wooden sticks at ground with relatively small slope angles, and avoided placing them near large boulders or hollows. Transplants were separated by a distance of about 20 cm. They were about 10 cm in diameter for *E. angustirete*, 5 cm in diameter for *B. lycopodiodes* and 3 cm for *H.seligeri*. Differences in transplant size between species were mainly due to differences in the availability of planting material.

To measure air and ground temperature, we installed two temperature loggers (ibuttons, DS1922) at each site. One logger was placed at the ground level underneath a moss-patch inside two plastic zip-bags and the other one was attached to the top of a wooden pole covered by a plastic cup, one meter above the ground. The temperature was recorded every half hour during the experimental period.

We extracted twelve different microclimatic variables from the temperature data for the period 29th of May until 28th of September 2011 (Table S1). Mean temperature and the mean diurnal temperature range were calculated for both air and ground temperature. Additionally, we derived the 5^th^ and 95^th^ percentiles of the minimum and maximum air and ground temperatures [18]. The 5^th^ percentile of the minimum temperatures and the 95^th^ percentile of the maximum temperatures were used to represent extreme cold and extreme warm conditions, respectively. The 95^th^ percentile of the minimum temperatures and the 5^th^ percentile of the maximum temperatures represent mild minimum and mild maximum temperatures, respectively. A high mild minimum temperature indicates that most nights of a site generally remain relatively warm, whereas a high mild maximum temperature indicates that most of the days remain relatively warm [18].

We retrieved sixteen additional environmental variables: altitude, basal area, broadleaved trees, canopy cover, distance to open ground, distance to the sea, ground vegetation, pH litter, pH soil, productivity (assessed using vegetation types, see below), relative elevation, shrubs, slope, solar radiation, tree age and tree height. Solar radiation (direct radiation plus diffuse radiation in kWh/m^2^) between the 29th of May and the 28th of September of 2011 was calculated for each site by the points solar radiation tool (spatial analyst extension), using default values (the uniform sky model, transmittivity = 0.5, proportion of diffuse radiation = 0.3) in ArcGIS Desktop 10.0. The input for the calculations was a digital elevation model (DEM) with a grid of 50×50 meter cells provided by Lantmäteriet (the Swedish mapping, cadastral and land registration authority, www.lantmateriet.se). Distance to the sea and distance to open ground was estimated by the shortest distance from the site midpoints to the coast (Figure 1) and to open ground respectively, using topographic maps and aerial photos in ArcGIS Desktop 10.0. As an estimate of productivity, we classified the vegetation within each site into one of six categories representing an increasing gradient in productivity (corresponding to the standard method of Hägglund & Lundmark [43]): (1) dwarf-shrubs *Calluna vulgaris* and *Empetrum nigrum*; (2) dwarf-shrub *Vaccinium vitis-idaea*; (3) dwarf-shrub *V. myrtillus*; (4) low herbs and dwarf-shrubs (*V. myrtillus*); (5) low herbs or (6) tall herbs. Nomenclature follows Mossberg and Stenberg [44] for vascular plants, Hill et al. [45] for mosses and Damsholt [46] for liverworts. See Table S1 and Text S1 for definitions and data collection methods on the remaining environmental variables, since they were only used to characterize north- and south-facing slopes and not included in the performance analyses (see below).

We calculated growth as the change in transplant size over the study for *B. lycopodioides* and *E. angustirete*. Transplant size was estimated from photographs taken at the start (30th of April until 28th of May 2011) and end of the experimental period (29th of September until 7th of October 2011), and placing a ruler along each transplant. We used the program GIMP 2.6.11 to delimit polygons of the living parts in each transplant photograph. We did not study growth of *H. seligeri* because it was restricted to grow on available pieces of wood. For all species we estimated the transplant vitality of the living parts (of the gametophyte) at the end of the experimental period according to the following scale: (1) <50% of the transplant was freshly green (vigorous); (2) 50–<95% of the transplant was freshly green; (3) ≥95% of the transplant was freshly green. The remaining parts of the transplants were brownish/pale and therefore not judged as vital. These categories were assumed to reflect the overall condition of each transplant, and to relate to vital rates such as growth and survival [47]. As a measurement of reproduction, we recorded the number of immature capsules at the first occasion and the capsules that had matured at the second occasion in the monoicous *H. seligeri*. We did not record more than a handful sporophytes on the two dioicous species *B. lycopodioides* and *E. angustirete*.

All necessary permits were obtained for the described field studies. Permissions were granted from the County administrative board of Västernorrland and from the landowners (Kramfors and Sollefteå municipalities, SCA, the Church of Sweden and private landowners). The field studies did not involve endangered or protected species.

### Data analyses

We examined among-site variation in six performance- variables: 1–2) mean growth and vitality of *B. lycopodioides*, 3–4) mean growth and vitality of *E. angustirete*, and 5–6) mean vitality and capsule maturation (proportion of capsules that matured) of *H. seligeri*. Growth and vitality of *E. angustirete* and vitality of *H. seligeri* were log-transformed before statistical analyses in order to improve model performance.

We tested if the six performance variables differed between north- and south facing slopes. Growth and vitality of *E. angustirete* and vitality of *H. seligeri* were normally distributed and t-tests were used. For *B. lycopodioides* vitality and *H. seligeri* capsule maturation, we used Wilcoxon rank-sum test because the compared groups did not conform to the assumption about normality. The Wilcoxon test of *H. seligeri* capsule maturation was performed with the proportions of the pooled number of capsules that matured per site. We used Bonferroni-adjusted p-values for the two tests on each species. Moreover, we compared environmental variables between north- and south-facing slopes. First, we performed a comparison of all measured environmental variables between the slope aspects by using a MANOVA with Pillai's trace test. Since we found an overall difference among aspect groups in environmental variables, we continued to explore differences among individual variables. For distance to open ground, distance to the sea, pH soil, productivity, tree age, slope, broadleaved trees, ground vegetation and shrubs we performed Wilcoxon rank-sum tests because the compared groups were not normally distributed. For the other environmental variables, we used Welch's t-tests. Canopy cover was arcsine-transformed before tests. Additionally, the relationships among the environmental variables were visualized through a PCA analysis using the package vegan (Figure S1). The variables were centered and scaled to unit variance. Otherwise, default settings were used.

To select microclimatic variables for models of bryophyte performance, we examined covariance patterns using Pearson's product-moment correlations. Many of the microclimatic variables were inter-correlated (Table S3). To keep problems associated with collinearity low while providing a reasonable representation of microclimatic variation, we chose the group of four variables with the lowest maximum correlation coefficient. The microclimatic variables selected according to these criteria were 1) extreme cold air temperature, 2) mild maximum air temperature, 3) extreme warm air temperature, and 4) diurnal ground temperature range. In addition to the microclimatic variables we included four variables that were assumed to influence individual performance: distance to the sea, distance to open ground, solar radiation and productivity. Distance to sea and distance to open ground were both log-transformed, since we might suspect that their influence on climatic conditions increases exponentially with decreasing distance [26], [34]. Among the eight environmental variables included in the analyses, the highest correlation coefficient was 0.65 (Pearson's product-moment correlation; Table S4).

To examine how the six performance variables did depend on these eight environmental variables, we used lasso regression (‘least absolute shrinkage and selection operator’; glmnet package in R). We performed lasso regression with gaussian error terms for the growth and vitality variables, whereas lasso regression with binomial error term was used for capsule maturation. For the latter regression, we used count data (number of capsules that matured out of total per site) instead of proportions. Lasso regression method works through shrinkage of the regression coefficients by using a cap on the sum of the absolute value of all the coefficients. In lasso, these coefficients are often shrunk exactly to zero, which is equal to removal of explanatory variables from a regression model. The glmnet package uses the penalty λΣ_j_|β_j_| to residual sum of square instead of, but equivalent to, constraining the sum of coefficients [48], [49]. Cross-validation with number of folds equal to sample size was performed to find the λ-value that minimized the residual sum of squares. Regression shrinkage methods such as lasso and ridge selection have been shown to perform better than subset selection using BIC when there are few observations per predictor [49]–[51]. Also considering the similarity between subset selection and stepwise regression, regression shrinkage methods should therefore be considered when there are few observations per predictor [51]. The residuals of the lasso-models were examined through plotting the corresponding linear regression models, in which they showed no particularly heteroscedasticity or skewness. Lastly, we checked for spatial autocorrelation among the performance variables through Mantel tests. We applied Bonferroni correction on the p-values for the two tests on each species and used proportions for capsule maturation. No spatial autocorrelation was found. From all analyses, samples with missing values were excluded. The analyses were done in R 3.0.2 [52].

## Results

### North- vs. south-facing slopes

**Figure 2:**
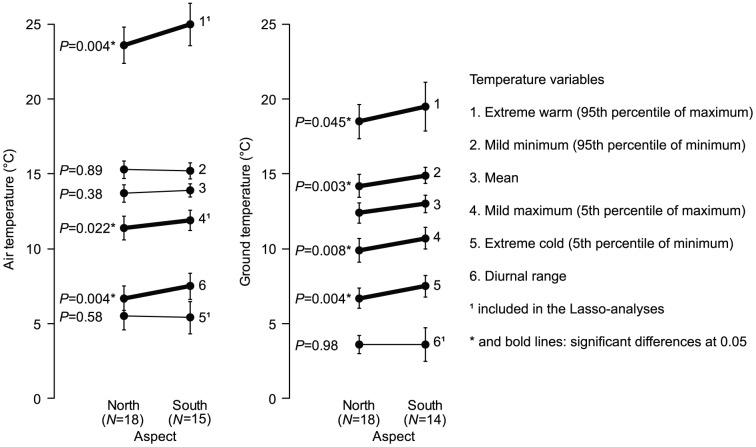
Comparison of ground and air temperatures (mean ±1 SD) between the north- and south-facing slopes. p-values were derived through Welch's t-tests.

There was an overall difference in the measured environmental variables between slope aspects (MANOVA, p = 0.015). Not all microclimatic variables did differ between the slopes, despite south-facing slopes receiving more incoming solar radiation than north-facing (Table 1). Largest differences were found for the extreme warm air temperature, the diurnal air temperature, the mild minimum ground temperature and the extreme cold ground temperature. South- and north-facing slopes differed more with regards to ground temperature variables than to air temperature (Figure 2; Table S2). Besides differences in several temperature variables, the south-facing slopes had on average taller trees and higher soil and litter pH than the north-facing slopes (Table S2).

**Table 1:** Comparison between south- and north-facing slopes of the environmental variables used in the performance analyses (except for microclimatic variables, see Table S2).

Variable category	Environmental variable	South	North	p-value
**Variable stratified to be similar between aspects**	Distance to open ground (m)	76 (10–372)	45 (10–130)	0.56
	Distance to the sea (km)	20 (0–51)	17 (0–51)	0.56
**Variable not stratified to be similar between aspects**	Solar radiation (kWh per square meter)	483 (454–505)	354 (274–411)	<0.001*
	Productivity (scale 1–6, see Materials and Methods)	4 (3–5)	4 (2–6)	0.10

For each environmental variable, mean values are presented at south- and north-facing slopes respectively. The minimum and maximum values are noted within the parentheses. For productivity, the median values are shown instead of the means. N was 15 at south-facing slopes and 18 at north-facing slopes. The p-values were derived from comparisons between north- and south-facing slopes through Welch's t-tests.

* Significance at the 5% level.

The PCA showed that many of the environmental variables were sorted along the north-south gradient. However, many other variables such as distance to the sea and the two minimum air temperatures were orthogonal to the north-south axis (Figure S1).

### Performance of the transplants

**Figure 3:**
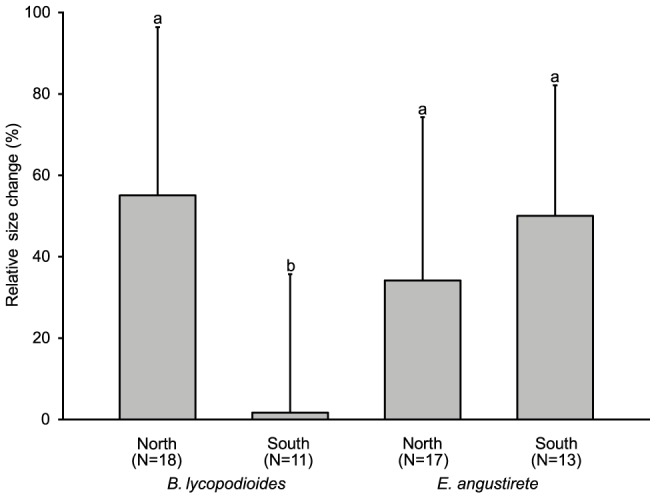
Mean growth (percentage ±1 SD) of *B. lycopodioides* and *E. angustirete* at north- and south-facing slopes. Significant differences (at p<0.05) between the two groups for each species are indicated with the symbols a–b.

The northerly distributed liverwort *B. lycopodioides* increased significantly more in size and had higher vitality at north-facing than at south-facing slopes (Figure 3; Table 2). The optimal lasso regression models of *B. lycopodioides* included negative effects of solar-radiation on both growth and vitality, while higher productivity resulted in higher vitality (Table 3).

**Table 2:** Comparison of the transplant performance between south- and north-facing slopes.

Performance variable	South	North	p-value	N (south)	N (north)
*B. lycopodioides* growth (%)	1 (−48–50)	55 (−24–144)	0.0024^b^ *	12	18
*B. lycopodioides* vitality	1.9 (1.0–2.7)	2.2 (2.0–3.0)	0.016* c	12	18
*E. angustirete* growth (%)	50 (−8–106)	34 (−50–93)	0.22^a^	13	17
*E. angustirete* vitality	2.0 (1.7–2.3)	2.0 (1.3–2.7)	1.00^a^	13	17
*H. seligeri* capsule maturation (%)	57 (10–91)	61 (13–100)	0.64^c^	15	18
*H. seligeri* vitality	2.1 (2.0–2.5)	2.1 (1.7–3.0)	0.98^b^	15	18

For the performance variables of growth and capsule maturation, percentage values are presented at south- and north-facing slopes respectively, while mean values are shown for vitality. The minimum and maximum values are noted within the parentheses. Values used in the comparison tests were mean values per site of log(final area/initial area) for growth, proportion of the pooled number of capsules that matured per site for capsule maturation, and mean values per site for vitalities of *B. lycopodioides*, *E. angustirete* (log-transformed) and *H. seligeri* (log-transformed). N indicates the number of north- and south-facing slopes in the analyses of which some were excluded due to missing values.

a p-values that were derived from Student's t-tests.

b p-values that were derived from Welch's t-tests.

c p-values that were derived from Wilcoxon rank-sum tests.

* Significant at the 2.5% level after Bonferroni correction for each species.

**Table 3:** Optimal lasso regression models of effects of environmental variables on the performance variables growth of *B. lycopodioides* and *E. angustirete*, and vitality of *B. lycopodioides* and *H. seligeri*.

Response variable	Penalty (λ)	R^2^	Intercept	Predictors and estimates
*B. lycopodioides* growth (log)	0.04	0.18	0.32	solar radiation −0.0006
*B. lycopodioides* vitality	0.08	0.21	2.38	productivity 0.08
				solar radiation −0.001
*E. angustirete* growth (log)	0.06	0.070	−0.17	mild maximum air temperature 0.02
*H. seligeri* vitality (log)	0.01	0.11	0.32	distance to the sea (log) 0.01

The environmental variables used in the analyses were: extreme cold air temperature, mild maximum air temperature, extreme warm air temperature, diurnal ground temperature range, distance to open ground, distance to the sea, solar radiation and productivity. As response variables, we used mean values per site of log(final area/initial area) for growth of *B. lycopodioides* and *E. angustirete*, and mean values per site for the vitalities of *B. lycopodioides* and *H. seligeri* (log-transformed). N was 29 for the response variables of *B. lycopodioides*, 29 for *E. angustirete* growth and 32 for *H. seligeri* vitality.

In the southern moss *E. angustirete*, there were no significant differences in growth or vitality between north and south-facing slopes (Figure 3; Table 2). The optimal lasso regression model for growth of *E. angustirete* included a positive effect of increasing mild maximum air temperature (Table 3), but did not identify any environmental variable related to variation in vitality.

There were no significant differences between north and south-facing slopes in vitality or capsule maturation of the southern moss *H. seligeri* (Table 2). The optimal lasso regression model identified a positive effect of distance to the sea on vitality of *H. seligeri* (Table 3). Variation in capsule maturation was not related to any environmental variable.

## Discussion

This study showed that aspect and microclimate were important in explaining the growth of two transplanted bryophyte species. However, the pattern was more complex than simply northern species performing better on northern slopes and in cold sites and southern species performing better on south facing slopes and in warm sites. This complexity may be explained by that not all microclimatic variables differed between aspects (cf. Figure 2; Table S2). Thus, even if the performance of a species is directly regulated by climatic variables it is not necessarily true that it will show different performance at north- vs. south-facing slopes.

Transplant performance of the northern liverwort *B. lycopodioides* was, as hypothesized, better at north-facing slopes. This is in agreement with this species' distribution pattern, since it has been found to be more abundant at north-facing than at south-facing slopes in the study area [41]. Poor individual growth might be a part of the explanation for low abundance of a species at its range limits, and has been found to coincide with low reproduction of trees at their range margins, and with either low reproduction or low survival of herbs transplanted beyond their altitudinal range limits [53]–[55]. Moreover, increased growth of individuals often leads to both higher survival and increased population growth rate [47], [56], [57].

Since effects of interspecific competition were not important in this study, the poor transplant performance of *B. lycopodioides* at south-facing slopes indicates that the species occurrences might be limited by abiotic factors, and that south-facing slopes are outside the species fundamental niche. The Lasso-model did not identify any effects of temperature variables on the growth or vitality of *B. lycopodioides* (Table 3), which imply that other abiotic factors could be important for its distribution pattern in the study area. It is probable that the south-facing slopes also in our study area are drier compared to north-facing, because they have larger temperature fluctuations (Figure 2), which implies less stable moisture conditions [20]. Therefore, it is possible that *B. lycopodioides* is more limited by drought than by high temperatures *per se* in this landscape. Also other studies of terrestrial plants in temperate Europe have revealed that climate, in particularly drought, frequently limit species ranges towards the equator [4], [58], [59]. Moreover, liverworts are generally considered more drought sensitive compared to mosses [60], [61], and consequently favored under moist conditions. Future studies should thus take into account variations in both ground and air humidity which may have as strong or stronger impact than temperatures *per se*.

One way of testing if a species is in equilibrium with current climatic conditions, and thereby if climatic conditions are range limiting, is to transplant individuals outside its range margins [5], [62]. For *E. angustirete* transplanted outside its range, the good performance suggests that most study sites lies within its climatic niche. This would imply that the species distribution is in disequilibrium with current climatic conditions and that microclimatic conditions are not the main limiting factors at the northern range margin of *E. angustirete*. This would thus represent a different pattern compared to that of several bryophytes in central Europe, which range margins towards colder regions are related to winter isotherms [63]. However, extreme climatic events often have been shown to increase mortality or physiological stress at range margins of different species [5], [64]. Long-term studies might therefore be necessary to conclude if species ranges are limited by rare climatic conditions, or if other factors, such as dispersal, are limiting. Transplant experiments in which individuals have been placed outside their range margins and performed well, have been carried out also for other species, e.g., forest herbs [65], [66]. In these studies, the species have been suggested to be dispersal limited and therefore in disequilibrium with climate. However, bryophytes have rather small spores which potentially can be wind-dispersed over long distances [67]. Therefore, dispersal is not likely to be a main limiting factor for bryophytes that often produce spore capsules. It is probable that at least some individual shoots in the often large populations of *E. angustirete* produce capsules over a growing-season, which would make dispersal limitation less important. Still, a species could be limited by interspecific competition, possibly also in combination with unfavorable environmental conditions such as low temperature or ground pH, thereby limiting its range [47], [68], [69].

Growth and survival of early life-stages in margin populations have been suggested to be important in determining range edges [5], [10], [70]. A study in which butterflies were transplanted outside their range margins showed that cool temperatures hampered the development of early life-stages. Therefore, unfavorable climatic conditions were suggested to directly limit the range margins of this species [71]. Further on, although transplanted adults of a crab species survived beyond their range margin, lab experiments suggested that low temperature were still limiting ranges through negative effects on larval development [72]. Analogously, climatic equilibrium could prevail in *E. angustirete* if climate limit the species range through its impact on early life-stages in spite of that adult transplants grew well.

Even if transplant performance of *E. angustirete* was relatively good, transplants were growing better at sites with high mild maximum air temperatures, which is in agreement with its geographical distribution towards warmer regions. Although the mild maximum air temperature was higher at south facing slopes, the performance did not significantly differ between north- and south-facing slopes. This could be explained by that also other climate-forcing factors such as altitude are important for the mild maximum air temperature. Furthermore, other temperature variables could influence transplant performance, but not all of them did differ between north- and south-facing slopes (Figure 2). Additionally, water availability could possibly be important for the transplant performance of *E. angustirete*. The transplants might have been water-saturated and active, and subsequently growing for longer periods at north-facing slopes due to longer lasting moisture. At south facing slopes, they might have been growing faster due to higher daytime temperatures that were closer to their temperature optimum. At the same time, their growth periods were probably shorter at south-facing slopes due to longer drought periods with water deficit. After such drought periods, the transplants should have been able to start growing quickly since *E. angustirete* is a rather desiccation-tolerant species [73]. This could have contributed to the roughly equal performance at both aspects, and its desiccation-tolerance might explain the relatively high vitality at both aspects in spite of the potentially drier environment at south-facing slopes.

The results for vitality and capsule maturation in *H. seligeri* suggest that the performance might not be related to its distribution, since also this species performed well on north-facing slopes and had higher vitality further inland which does not coincide with its realized niche. Furthermore, no microclimatic influence on capsule maturation was found.

### Conclusion

This study provided equivocal support for that the species geographic distribution patterns reflected their individual performance in relation to microclimatic gradients. The results for *B. lycopodioides* was consistent with that species with more northern distribution perform better at north-facing slopes. However, both the southern species performed well outside their range and only *E. angustirete* was significantly affected by microclimate. By understanding climatic influence on all vital rates and, thus, population growth rate at range margins, we should be able to predict the influence of a warming climate on species potential distributions. The complex microclimatic gradients across the landscape make it difficult to rely only on aspect as an explanatory variable for performance. This is particularly important in topographically complex landscapes where larger differences between ground and air temperatures might be suspected. It is important to bear in mind that our study tests hypotheses for the three different species and does not allow any broader inferences regarding differences between northern vs. southern species, or mosses vs. liverworts. Still, well-replicated studies on three different species provide important evidence of that relationships between distributions and environmental factors might be much more complex than often anticipated.

## Supporting Information

Figure S1 **PCA analysis of the environmental variables related to sites.** Light-grey points represents north-facing slopes, dark-grey triangles represents south-facing slopes. Black arrows marks environmental variables as follows: A = Altitude (m), Ag = Tree age (years), B = Broadleaved trees (%), Ba = Basal area (m3), C = Canopy cover (%), G = Ground vegetation (cm), H = Tree height (m), N = North-facing slopes, O = Distance to open ground (log(m)), Phl = pH litter, Phs = pH soil, Re = Relative elevation (m), S = South-facing slopes, Se = Distance to the sea (log(km)), Sh = Shrubs (%), Sl = Slope (°), T5maa = Mild maximum air temperature (°C), T5mag = Mild maximum ground temperature (°C), T5mia = Extreme cold air temperature (°C), T5mig = Extreme cold ground temperature (°C), T95maa = Extreme warm air temperature (°C), T95mag = Extreme warm ground temperature (°C), T95mig = Mild minimum air temperature (°C), T95mig = Mild minimum ground temperature (°C), Tma = Mean air temperature (°C), Tmg = Mean ground temperature (°C), Tra = Diurnal air temperature range (°C), Trg = Diurnal ground temperature range (°C), P = productivity.
(EPS)

Table S1 **Measured environmental variables.**
(DOC)

Table S2 **Environmental variables not included in the performance analyses and microclimatic variables.** These are compared between south- and north-facing slopes, for which mean, minimum and maximum values are presented.
(DOC)

Table S3 **Pearson product-moment correlation analyses between the microclimatic variables.**
(DOCX)

Table S4 **Pearson product-moment correlation analyses between the environmental variables used in lasso regression.**
(DOC)

Text S1 **Methods for collecting additional environmental data that were not included in the performance analyses (see also Tables S1 and S2).**
(DOC)
