# Supplementary material for: Performance of Forest Bryophytes with Different Geographical Distributions Transplanted across a Topographically Heterogeneous Landscape
Source: PLoS One. 2014 Nov 11;9(11):e112943. doi: 10.1371/journal.pone.0112943 (PMC4227873; doi:10.1371/journal.pone.0112943)
Supplement: Table S1 — Measured environmental variables. (DOC) [file pone.0112943.s002.doc]

| **Variable category** | **Environmental variable** | **Definition** |
| --- | --- | --- |
| **1. Microclimatic variable** | *Extreme cold air temperature (°C)* | *5th percentile of the daily, minimum air temperature* |
|  | Mild minimum air temperature (°C) | 95th percentile of the daily, minimum air temperature |
|  | *Mild maximum air temperature (°C)* | *5th percentile of the daily, maximum air temperature* |
|  | *Extreme warm air temperature (°C)* | *95th percentile of the daily, maximum air temperature* |
|  | Extreme cold ground temperature (°C) | 5th percentile of the daily, minimum ground temperature |
|  | Mild minimum ground temperature (°C) | 95th percentile of the daily, minimum ground temperature |
|  | Mild maximum ground temperature (°C) | 5th percentile of the daily, maximum ground temperature |
|  | Extreme warm ground temperature (°C) | 95th percentile of the daily, maximum ground temperature |
|  | Mean air temperature (°C) | mean air temperature |
|  | Diurnal air temperature range (°C) | mean, diurnal, air temperature range |
|  | *Diurnal ground temperature range (°C)* | *mean, diurnal, ground temperature range* |
|  | Mean ground temperature (°C) | mean ground temperature |
| **2. Additional environmental variable** | Altitude (m) | altitude at square midpoint |
|  | Basal area (m^3^) | total basal area of tree layer |
|  | Broadleaved trees (%) | percentage broadleaved trees |
|  | Canopy cover (%) | percentage canopy cover above square midpoint |
|  | *Distance to open ground (m)* | *shortest distance to open ground from square midpoint* |
|  | *Distance to the sea (km)* | *shortest distance to the sea from square midpoint* |
|  | Ground vegetation (cm) | mean height of field layer inside square |
|  | pH litter | pH of litter inside square |
|  | pH soil | pH of soil inside square |
|  | *Productivity (scale, see methods)* | *soil productivity, 1 - 6, inside square* |
|  | Shrubs (%) | percentage shrub cover inside square |
|  | Slope (°) | slope inclination of moss patches |
|  | *Solar radiation (kWh per m^2^)* | *total incoming solar radiation, square midpoint* |
|  | Tree age (years) | estimated age of mature tree layer |
|  | Tree height (m) | height of a type tree within 10 m from square midpoint |
|  | Relative elevation (m) | altitude, square midpoint, relative to lowest altitude within 500 m |

**Table S1: Measured environmental variables.**

The variables in italics were chosen for the performance analyses
