# Supplementary material for: Performance of Forest Bryophytes with Different Geographical Distributions Transplanted across a Topographically Heterogeneous Landscape
Source: PLoS One. 2014 Nov 11;9(11):e112943. doi: 10.1371/journal.pone.0112943 (PMC4227873; doi:10.1371/journal.pone.0112943)
Supplement: Table S2 — Environmental variables not included in the performance analyses and microclimatic variables. These are compared between south- and north-facing slopes, for which mean, minimum and maximum values are presented. (DOC) [file pone.0112943.s003.doc]

**Table S2: Environmental variables not included in the performance analyses and microclimatic variables: These are compared between south- and north-facing slopes, for which mean, minimum and maximum values are presented.**

| **Variable category** | **Environmental variable** | **South** | **North** | **p-value** |
| --- | --- | --- | --- | --- |
| **Microclimatic variable** | Extreme cold air temperature (°C) | 5.4 (3.7-6.6) | 5.5 (3.6-7.1) | 0.58 |
|  | Mild minimum air temperature (°C) | 15.2 (14.2-16.1) | 15.3 (14.2-16.1) | 0.89 |
|  | Mild maximum air temperature (°C) | 11.9 (10.6-12.7) | 11.4 (10.1-12-6) | 0.020* |
|  | Extreme warm air temperature (°C) | 25.0 (22.7-27.5) | 23.6 (22.0-26.1) | 0.004* |
|  | Extreme cold ground temperature (°C) | 7.5 (6.6-8.7) | 6.7 (5.6-8.1) | 0.004* |
|  | Mild minimum ground temperature (°C) | 14.9 (14.2-16.1) | 14.2 (12.6-15.2) | 0.003* |
|  | Mild maximum ground temperature (°C) | 10.7 (9.8-12.0) | 9.9 (8.6-11.7) | 0.008* |
|  | Extreme warm ground temperature (°C) | 19.5 (17.2-22.6) | 18.5 (16.7-20.6) | 0.045* |
|  | Mean air temperature (°C) | 13.9 (13.3-14.4) | 13.7 (12.6-14.7) | 0.38 |
|  | Mean ground temperature (°C) | 13.0 (12.4-14.2) | 12.4 (11.2-13.4) | 0.010* |
|  | Diurnal air temperature range (°C) | 7.5 (6.2-8.8) | 6.6 (5.3-7.9) | 0.004* |
|  | Diurnal ground temperature range (°C) | 3.6 (2.4-6.8) | 3.6 (2.5-4.5) | 0.98 |
| **Variable stratified to be similar between aspects** | Altitude (m) | 142 (42-279) | 173 (15-335) | 0.38 |
|  | Relative elevation (m) | 52 (16-98) | 63 (15-100) | 0.14 |
|  | Slope (°) | 13 (5-27) | 11 (3-20) | 0.79^b^ |
|  | Tree age (years) | 86 (50-100) | 88 (50-150) | 1.00^b^ |
| **Variable not stratified to be similar between aspects** | Basal area (m^3^) | 23 (14-37) | 20 (10-32) | 0.13 |
|  | Canopy cover (%) | 73 (59-86) | 68 (38-80) | 0.10 |
|  | Broadleaved trees (%) | 21 (0-59) | 19 (0-54) | 0.48^b^ |
|  | Ground vegetation (cm) | 5.5 (0.5-10.9) | 6.4 (1.0-12.4) | 0.49^b^ |
|  | pH litter | 5.3 (4.8-5.8) | 5.1(4.6-5.5) | 0.010* |
|  | pH soil | 4.7(4.1-5.5) | 4.2(3.9-4.6) | 0.010*^b^ |
|  | Shrubs (%) | 3 (0-25) | 4 (0-20) | 0.31^b^ |
|  | Tree height (m) | 21 (11-29) | 17 (10-24) | 0.010* |

N were 15 for south-facing slopes, except for ground temperatures where N was 14. For north-facing slopes, N were 18. The p-values were derived from comparisons of north-facing slopes with south-facing slopes. Canopy cover was arcsine-transformed.

^b^ P-values that were derived from Wilcoxon rank-sum tests (otherwise Welch’s t-test was used)

* Significance at the 5 % level
