# Supplementary material for: Performance of Forest Bryophytes with Different Geographical Distributions Transplanted across a Topographically Heterogeneous Landscape
Source: PLoS One. 2014 Nov 11;9(11):e112943. doi: 10.1371/journal.pone.0112943 (PMC4227873; doi:10.1371/journal.pone.0112943)
Supplement: Table S3 — Pearson product-moment correlation analyses between the microclimatic variables. (DOCX) [file pone.0112943.s004.docx]

**Table S3: Pearson product-moment correlation analyses between the microclimatic variables.**

| **Temperature variable** | **Diurnal ground temperature range** | **Diurnal air temperature range** | **Air temperature** | **Ground temperature** | **Extreme cold ground temperature** | **Mild minimum ground temperature** | **Mild maximum ground temperature** | **Extreme warm ground temperature** | **Extreme cold air temperature** | **Mild minimum air temperature** | **Mild maximum air temperature** | **Extreme warm air temperature** |
| --- | --- | --- | --- | --- | --- | --- | --- | --- | --- | --- | --- | --- |
| Diurnal ground temperature range | ***** | 0.26 | -0.15 | 0.32 | -0.25 | 0.060 | 0.43 | 0.75* | 0.10 | -0.18 | -0.14 | 0.28 |
| Diurnal air temperature range | 0.15 | ***** | 0.20 | 0.22 | 0.13 | 0.14 | 0.21 | 0.28 | -0.61* | -0.33 | 0.28 | 0.85* |
| Air temperature | 0.40 | 0.26 | ***** | 0.63* | 0.69* | 0.63* | 0.58* | 0.14 | 0.51* | 0.80* | 0.88* | 0.31 |
| Ground temperature | 0.08 | 0.23 | <0.001* | ***** | 0.77* | 0.89* | 0.91* | 0.69* | 0.40 | 0.52* | 0.70* | 0.34 |
| Extreme cold ground temperature | 0.16 | 0.49 | <0.001* | <0.001* | ***** | 0.80* | 0.65* | 0.24 | 0.45 | 0.60* | 0.76* | 0.26 |
| Mild minimum ground temperature | 0.76 | 0.46 | <0.001* | <0.001* | <0.001* | ***** | 0.76* | 0.57* | 0.44 | 0.58* | 0.67* | 0.30 |
| Mild maximum ground temperature | 0.02* | 0.24 | 0.001* | <0.001* | <0.001* | <0.001* | ***** | 0.67* | 0.41 | 0.49 | 0.67* | 0.31 |
| Extreme warm ground temperature | <0.001* | 0.12 | 0.43 | <0.001* | 0.19 | 0.001* | <0.001* | ***** | 0.14 | 0.11 | 0.15 | 0.46 |
| Extreme cold air temperature | 0.57 | <0.001* | 0.003* | 0.020* | 0.010* | 0.013* | 0.021* | 0.44 | ***** | 0.83* | 0.36 | 0.29 |
| Mild minimum air temperature | 0.32 | 0.060 | <0.001* | 0.002* | <0.001* | 0.001* | 0.004* | 0.54 | <0.001* | ***** | 0.66* | 0.080 |
| Mild maximum air temperature | 0.43 | 0.12 | <0.001* | <0.001* | <0.001* | <0.001* | <0.001* | 0.41 | 0.039* | <0.001* | ***** | 0.25 |
| Extreme warm air temperature | 0.12 | <0.001* | 0.08 | 0.050* | 0.15 | 0.01* | 0.080 | 0.008* | 0.11 | 0.66 | 0.17 | ***** |

Upper diagonal part contains correlation coefficients, while lower diagonal part contains corresponding p-values. N were 15 for south- and 18 for north-facing slopes.

* Significance at the 5 % level and correlation-values above 0.5
