# Supplementary material for: Performance of Forest Bryophytes with Different Geographical Distributions Transplanted across a Topographically Heterogeneous Landscape
Source: PLoS One. 2014 Nov 11;9(11):e112943. doi: 10.1371/journal.pone.0112943 (PMC4227873; doi:10.1371/journal.pone.0112943)
Supplement: Table S4 — Pearson product-moment correlation analyses between the environmental variables used in lasso regression. (DOC) [file pone.0112943.s005.doc]

**Table S4: Pearson product-moment correlation analyses between the environmental variables used in lasso regression.**

| **Environmental variable** | **Distance to open ground (log)** | **Distance to the sea (log)** | **Solar radiation** | **Productivity** | **Extreme cold air temperature** | **Mild maximum air temperature** | **Extreme warm air temperature** | **Diurnal ground temperature range** |
| --- | --- | --- | --- | --- | --- | --- | --- | --- |
| Distance to open ground (log) | ***** | 0.12 | 0.23 | -0.040 | 0.005 | -0.36 | 0.11 | 0.25 |
| Distance to the sea (log) | 0.51 | ***** | 0.28 | 0.17 | -0.65* | -0.34 | 0.54 | 0.070 |
| Solar radiation | 0.19 | 0.11 | ***** | 0.32 | 0.22 | 0.25 | 0.62* | 0.10 |
| Productivity | 0.84 | 0.36 | 0.080 | ***** | 0.22 | 0.00 | 0.040 | -0.42 |
| Extreme cold air temperature | 0.98 | <0.001* | 0.22 | 0.21 | ***** | 0.36 | -0.29 | -0.10 |
| Mild maximum air temperature | 0.040* | 0.060 | 0.17 | 1.00 | 0.040* | ***** | 0.25 | -0.14 |
| Extreme warm air temperature | 0.55 | 0.001* | <0.001* | 0.82 | 0.11 | 0.17 | ***** | 0.28 |
| Diurnal ground temperature range | 0.17 | 0.69 | 0.57 | 0.020* | 0.57 | 0.43 | 0.12 | ***** |

Upper diagonal part contains correlation coefficients, while lower diagonal part contains corresponding p-values.

* Significance at the 5 % level and correlation-values above 0.5
