# Supplementary material for: Performance of Forest Bryophytes with Different Geographical Distributions Transplanted across a Topographically Heterogeneous Landscape
Source: PLoS One. 2014 Nov 11;9(11):e112943. doi: 10.1371/journal.pone.0112943 (PMC4227873; doi:10.1371/journal.pone.0112943)
Supplement: Text S1 — Methods for collecting additional environmental data that were not included in the performance analyses (see also Tables S1 and S2). (DOC) [file pone.0112943.s006.doc]

**Text S1: Methods for collecting additional environmental data that were not included in the performance analyses (see also Tables S1 and S2).**

We measured the basal area with a relascope from the site midpoints to get the proportion of each tree species. One meter above the ground, also from the site midpoints, we took photographs of the sky which were used to estimate the canopy cover. We analyzed the photographs with ImageJ v. 1.46 which converted these to black and white binary images. Then the proportion of white pixels was calculated to retrieve the percentage canopy cover.

We used a digital elevation model DEM (50 x 50 meter grid cells) provided by Lantmäteriet (the Swedish mapping, cadastral and land registration authority, www.lantmateriet.se) for calculations of the altitude and the relative elevation of the site midpoints. We calculated the relative elevation as the difference between the site altitude and the lowest altitude within a radius of 500 meters from the site midpoint [18,24]. A low value indicates that the site potentially is a location where cold air could pool from the surroundings [18].

We used an inclinometer to measure the slope inclination for the transplant patches. The inclinometer was also used when we measured the height of a type tree within 10 meters from the site midpoint, i.e. a tree which could represent the mature tree layer. Within 10 meters, we also estimated the mean tree age of the mature tree layer. Further on, we measured the height of the field layer vegetation (cm) with a ruler at fourteen or more randomly chosen spots within each site. Also, we estimated the percentage shrub cover at each site and collected soil samples from its 4 quadrants, from the surface down to 10 cm. We blended these into one soil sample per site. Using the same procedure, we also obtained litter samples. The samples were dried and then frozen for 6-12 months. After the samples had been defrosted, we measured pH for both the soil and litter samples with a pH-meter in the lab according to the standard SS-ISO 10 390.
